# Responses of Endothelial Progenitor Cells to Chronic and Acute Physical Activity in Healthy Individuals

**DOI:** 10.3390/ijms25116085

**Published:** 2024-05-31

**Authors:** Marta Tkacz, Katarzyna Zgutka, Patrycja Tomasiak, Maciej Tarnowski

**Affiliations:** 1 Department of Physiology in Health Sciences, Faculty of Health Sciences, Pomeranian Medical University in Szczecin, Zolnierska 48, 70-210 Szczecin, Poland; 2 Institute of Physical Culture Sciences, University of Szczecin, 70-453 Szczecin, Poland

**Keywords:** EPCs, CEPCs, chronic physical activity, acute physical activity, mobilisation of EPCs, CEPCs assessment

## Abstract

Endothelial progenitor cells (EPCs) are circulating cells of various origins that possess the capacity for renewing and regenerating the endothelial lining of blood vessels. During physical activity, in response to factors such as hypoxia, changes in osmotic pressure, and mechanical forces, endothelial cells undergo intense physiological stress that results in endothelial damage. Circulating EPCs participate in blood vessel repair and vascular healing mainly through paracrine signalling. Furthermore, physical activity may play an important role in mobilising this important cell population. In this narrative review, we summarise the current knowledge on the biology of EPCs, including their characteristics, assessment, and mobilisation in response to both chronic and acute physical activity in healthy individuals.

## 1. Introduction

The endothelium is a critical element of a functional, healthy vascular system. Considered to be an organ in itself, the blood vasculature supports all other organs by transporting respiratory gases, nutrients, metabolic waste, and blood cellular elements throughout the organism’s tissues. Endothelial cells (ECs) form a monolayer that lines the inner surfaces of blood vessels, serving as a barrier through which oxygen, nutrients, and solutes are transported. Beyond this barrier function, ECs also play numerous other roles of great importance to physiological body functioning. These include mediating the traffic of white blood cells into and out of the bloodstream, facilitating the adhesion and activation of platelets, regulating vasomotor tone, maintaining vascular integrity, and releasing tissue-specific growth factors essential for tissue homeostasis and metabolism [1,2,3,4,5]. Under normal conditions, the healthy vascular endothelium produces and secretes substances that modulate vascular physiology and protect the vessel wall from inflammatory cell infiltration, thrombus formation, and vascular smooth muscle cell proliferation, thus helping to maintain stem cell homeostasis [4].

Endothelial dysfunction and loss of the endothelial lining are common to all major cardiovascular diseases (CVDs) [6]. Immunological, haemodynamic, and biochemical alterations cause disturbances in endothelial function. Platelets maintain the integrity of the endothelium, and the endothelium in turn releases nitric oxide and prostacyclin to keep platelets in a resting state. However, in pathologic conditions such as hyperlipidaemia, hyperglycaemia, and hypertension, the ability of the vascular endothelium to produce vasodilatory and anti-adhesion molecules is impaired. Additionally, the production of vasoconstrictor, pro-adhesion, and pro-thrombotic molecules is increased, leading to elevated vascular tone, enhanced cell adhesion, proliferation of smooth muscle cells in the vessel wall, and an increased propensity toward thrombosis [6,7,8,9].

Like other organs and tissues, the endothelial layer undergoes degeneration and regeneration. As mentioned above, EC loss plays a critical role in the pathogenesis of multiple diseases. The scientific community has recently focused on the development of strategies to enhance rapid recovery of the EC layer while preserving its functionality and integrity. These efforts include research into stem cells capable of endothelial regeneration [10,11].

The discovery of endothelial progenitor cells (EPCs) has been credited to Asahara et al. [12], who identified a stem cell-like population (CD34^+^) in adult peripheral blood capable of eliciting postnatal vasculogenesis and endogenous neovascularisation of ischaemic tissues [13,14,15]. Moreover, the number and functionality of circulating EPCs (CEPCs) reflect the endogenous regenerative potential of the EC layer. These parameters decline in patients with cardiovascular disease and decrease in parallel with the ageing process [6,16,17,18]. We can envisage that upon endothelial damage, EPCs are mobilised from the bone marrow or other reservoirs and follow chemokine gradients to sites of endothelial injury, where they assist in endothelial repair. This phenomenon may be also named as endothelial turnover, which is specifically important in ischemic tissues. Stem cells within the bone marrow usually exist in a quiescent state, and specific signals stimulate them to differentiate and mobilise into the systemic circulation. Exercise training or physical activity (PA) is an important physiological stimulus for stem cell mobilisation; it improves vascular functionality and increases the number of CEPCs in healthy individuals and patients with serious conditions such as cardiovascular disease, chronic kidney disease, or cancer [10,11,19,20]. Hence, it increases the rate of endothelial turnover. This process is highly complicated and involves the coordination of a myriad of humoral factors such as cytokines, hormones, chemokines, and their receptors as well as cascades of intracellular signalling [11,20,21].

Thus, we can envisage that one of the ways to maintain vascular health and the regeneration of the endothelium is regular PA. Physical activity is widely recognised as a crucial intervention method and element of a healthy lifestyle. Regular PA yields numerous health benefits on multiple levels, including psychological, physiological, and molecular [22,23]. Consistent with this, reductions in PA limit the number of selected populations with stem cell-like characteristics capable of tissue regeneration [24]. Hence, PA may be a helpful tool to counteract cell proliferation decline with ageing or disease and increase the regenerative potential of the endothelium [25]. Specifically, aerobic exercise has been recommended for maintaining cardiovascular health. Such exercise increases the number of EPCs in the bloodstream [25,26,27], and aerobic PA has been proposed to foster endothelial regeneration by its positive effects on EPCs [28,29,30].

In this review, we examine the influence of PA on the EPCs compartment, the mobilisation of EPCs secondary to PA, and the potential applications of these cells in regenerative medicine.

## 2. Characterisation of EPCs

EPCs are precursors of ECs circulating in peripheral blood, and they are derived from the bone marrow and vascular niches. EPCs promote endothelial repair and neovascularisation and restore endothelial function. We may conclude that these circulating progenitors have hugely important roles in maintaining endothelial integrity and cardiovascular homeostasis [31,32].

Since the discovery of EPCs, many scientific studies have focused on their biology, culture assays, surface markers, origin, and differentiation hierarchy. This research has included both translational animal studies and clinical observations. However, the definition, origin, capabilities, and biological characteristics of these cells are highly controversial.

A self-renewing subpopulation of cells with stem/progenitor properties is needed to maintain endothelial homeostasis. These cells must be able to form and regenerate blood vessels in an organ-specific manner. Studies involving electron microscopy have shown significant EC turnover in various locations throughout the body, including the brain, liver, and pancreas, with blood vessels being described as mosaic structures consisting of young and aged ECs [33,34]. The number of EPCs in the circulation and peripheral tissues is very low [35]; therefore, their mobilisation from the bone marrow is a crucial step in promoting endothelial repair following physiological or pathological injury. In addition to the previously mentioned pathological mechanisms that disrupt endothelial function, an intense bout of PA induces abrupt shifts in blood pressure, osmotic pressure, mechanical stress, oxidative load, hypoxia, and pure shear stress. These factors collectively disturb endothelial function while also stimulating repair mechanisms, such as the mobilisation and proliferation of EPCs [25,30].

The process of EPC mobilisation has not yet been fully explored. When the body receives hypoxia signals, hypoxia-induced factor-1 (HIF-1) increases the expression of a variety of EPCs that activate angiogenic factors, including vascular endothelial growth factor (VEGF), stromal cell-derived factor-1 (SDF-1), monocyte chemoattractant protein-1 (MCP-1), insulin-like growth factor-1 (IGF-1), and erythropoietin (EPO) [36,37,38]. These factors induce EPCs to enter the peripheral blood. Because of the action of certain cytokines such as VEGF or SDF-1, EPCs migrate to the injured site and secrete matrix metalloproteins (MMPs), growth factors (hepatocyte growth factor (HGF), granulocyte colony-stimulating factor (G-CSF), and granulocyte–macrophage colony-stimulating factor (GM-CSF)), which promote the aggregation of CEPCs. Importantly, this paracrine activity is an important mechanism by which EPCs promote vascular repair [38]. The appearance of EPCs in the peripheral circulation signifies the activation of regenerative processes across the vascular system that eventually promote endothelial repair, neoangiogenesis, and the rescue of endothelial functions [38].

The endothelial regeneration promoted by EPCs is achieved either by their incorporation into blood vessels and differentiation into mature ECs or by stimulation of mature ECs to proliferate via a paracrine mechanism. The concept of CEPCs and their regenerative potential is grounded in this idea; however, it remains a topic of discussion within the scientific community [7,10,34].

Studies have shown that CEPCs do not represent a single cell type but rather a heterogenous population [39], the origins of which remain controversial. A recent study of patients who underwent sex-mismatched bone marrow transplantations showed that CEPCs do not originate entirely from the bone marrow but are also derived from niches in the inner walls of blood vessels [40].

Our current understanding is that EPCs comprise two distinct populations: haematopoietic EPCs (early EPCs) and non-haematopoietic EPCs (late EPCs). Haematopoietic EPCs express the three cell membrane surface markers CD133, CD34, and VEGF receptor-2 (VEGFR-2) (also termed kinase domain region (KDR)) and are unable to generate true ECs. However, they can differentiate into monocytic cells that exhibit some endothelial markers and exert pro-angiogenic effects in vivo through a paracrine mechanism [41,42]. These CEPCs, also termed circulating angiogenic cells (CACs) or EC colony-forming units (EC-CFUs) [39,43,44], have the ability to secrete a variety of cytokines, growth factors, lipids, and extracellular matrix proteins, providing nutritional and anti-apoptotic support for a variety of other cells with regenerative potential (e.g., ECs, cardiomyocytes, mesenchymal stem cells (MSCs), and neural stem cells (NSCs)) [36,38,45,46]. This paracrine production includes the secretion of cytokines, growth factors, and chemokines (including VEGF, SDF-1, platelet-derived growth factor (PDGF), and G-CSF) that promote angiogenesis and endothelial proliferation and migration [46,47,48]. Moreover, CEPCs can transmit a variety of signals through extracellular vesicles, apoptotic bodies, microvesicles, and exosomes [49].

Non-haematopoietic EPCs, also termed endothelial colony-forming cells (ECFCs) and outgrowth ECs, originate from CD34^+^/CD45^−^ progenitors mainly located in the blood vessel walls of the microvasculature. They have also been found in the human placenta [25]. ECFCs can generate large amounts of mature and functionally competent ECs both in vitro and in vivo [39,43,44,50]. They are characterised by remarkable clonogenic potential and postnatal vascularisation ability in vivo [51]. In vitro, they form capillary-like tubes in extracellular matrix gels, and they play a role in neovascularisation under ischaemic conditions and in re-endothelialisation upon endothelial injury [50]. Unlike haematopoietic EPCs, these cells seem capable of directly forming new vasculature, show high proliferative potential, and possess the capacity to differentiate into mature ECs [52]. Although evidence suggests that ECFCs arise from a vascular niche, their origin and specific location as resident ECs within the vascular wall in vivo remains to be proven. ECFCs have been shown to significantly contribute to vascular regeneration of ischaemic tissues in the heart, brain, retina, and limbs [34,53], making them promising future targets for cell therapy. Although their in vitro and in vivo angiogenic potentials greatly differ, both early and late EPCs are essential in the processes of endothelial repair and regeneration [54].

In this review, we report on current knowledge regarding the effects of long- and short-term chronic exercise on CEPC counts in healthy subjects. We performed a literature review using widely available databases, including PubMed, Web of Science, and Google Scholar. We searched for articles using the following keywords and combinations thereof: ‘physical activity’, ‘endothelial progenitor cells’, ‘exercises’, ‘number of endothelial progenitor cells’, ‘quantification of endothelial progenitor cells’, and ‘functionality of endothelial progenitor cells’. The search was completed in March 2024.

## 3. CEPCs Assessment Methods

The physiological role of EPCs in the repair of the injured endothelium makes these cells a promising target for multiple therapeutic strategies [13,15]. Numerous studies have shown the mobilisation of EPCs into the bloodstream following PA; therefore, PA may constitute an important preventive factor that attenuates the ageing process [3]. However, because of the different ways in which EPCs were assessed, the results of these studies cannot always be directly compared [55]. EPCs are characterised by more than 10 known phenotypes in the literature, making it difficult to relate the results of different studies Table 1.

In addition to the EPCs markers listed in Table 1, these cells also express other important molecules: von Willebrand factor (VWF), vascular endothelial cadherin (VE-cadherin, also known as CD144), CD38, endoglin (CD105), CXCR4 (CD184), fibroblast growth factor receptor (FGFR), c-kit, and angiopoietin 1 receptor precursor [also known as tunica intima EC kinase (Tie2/TEK)] [66].

The most widely used methods for EPCs analysis are clonogenic/CFU assays and flow cytometry. In general, clonogenic tests are performed by plating peripheral blood mononuclear cells (PBMCs) and presenting the data as the number of colonies (EC-CFUs) per well [55,67]. Several methods are available to assess the clonogenic potential of isolated EPCs. In one method, the CFU-Hill assay, early colony formation is assessed. After 2 days of culture on a fibronectin-coated dish, non-adherent cells are collected and cultured for an additional 9–10 days [55,62]. Further modifications of this method have allowed for the detection and differentiation of early and late colonies of EPCs. However, it has been emphasised that even single-cell cultures subjected to strict selection may still be susceptible to ‘reprogramming’ in vitro due to artificial environmental conditions [67].

Multi-colour staining enables the analysis of various endothelium-associated markers and stem cell markers by flow cytometry. However, this increases the diversity of the results and makes their direct comparison more difficult. Van Craenenbroeck et al. used different gating strategies for whole blood and PBMCs and demonstrated that each protocol was highly reproducible. However, agreement between EPCs quantification methods was poor, which may explain the apparent discrepancies in the literature [55]. The existence of different cellular phenotypes is certainly the main cause of these inconsistencies. In addition, mature ECs and haematopoietic stem/progenitor cells can also express CD34 and VEGFR-2 [66], which are typical EPCs markers.

Results from various studies have shown that peripheral blood and bone marrow-derived CD34+ cells can behave like ECs [66]. Case et al. isolated CD34+/AC133+/VEGFR-2+ cells from both human umbilical cord blood and G-CSF-mobilised peripheral blood and found that these cells did not give rise to EPCs and were devoid of vessel-forming activity [58]. Further, using immunoselection, the authors found that CD34+/CD45+ cells formed haematopoietic progenitor cells (HPCs) but not EPCs, whereas CD34+/CD45− cells formed EPCs but not HPCs [58]. Moreover, another series of studies involving fluorescence-activated cell sorting analysis revealed the presence of CD34+ and CD34- subpopulations among CD133+ progenitor cells. CD34−/CD133+ progenitors were found to differentiate into CD34+/CD133+ EPCs. These data indicate that the CD34−/CD133+ EPCs subpopulation is functionally more potent with respect to homing and vascular repair than the CD34+/CD133+ EPCs population [68]. Taken together, these contradictory results suggest that only a portion of CD34+ cells represent true EPCs and that EPCs are likely derived from a subpopulation of CD34+ cells [66]. Considering that CD34 and VEGFR-2 can also be expressed by mature ECs, more markers, such as CD133, are needed to identify and characterise EPCs [66].

Parallel analysis of several methods used for CEPCs assessment showed that the number of CFUs was not correlated with the number of CD34+/KDR+ or CD34+/CD133+/KDR+ cells and was negatively correlated with the number of CD34+/CD133+ cells [69]. However, the numbers of CD34+/KDR+ and CD34+/CD133+/KDR+ cells were correlated with the serum level of VEGF. Moreover, the number of CD34+/KDR+ EPCs adhering to fibronectin was correlated with the number of CFUs and not with either of the EPCs membrane markers. These findings show that the number of CFUs is correlated with the functional properties of EPCs [69]. This may indicate that a CD34+/KDR+ status is more appropriate for the definition of CEPCs and that the number of CFUs is more likely to reflect their ability to proliferate [69]. In the same study, the colonies were subjected to immunofluorescent phenotyping to detect the expression of ECs markers (anti-Tie2, anti-foetal liver kinase-1, and anti-CD31), in addition to staining with 1,1′-dioctadecyl-3,3,3′,3′-tetramethylindocarbocyanine perchlorate-acetylated low-density lipoprotein and lectin BS-1. This analysis confirmed that the vast majority of cells within the CFUs were indeed endothelial in nature [69].

Although each method has its drawbacks, the most objective technique is likely to be obtaining the count of differentially cultured EPCs by flow cytometry together with detecting specific markers of mature ECs (CD146 and vascular endothelial cadherin) [70]. Shantsila et al. found significant fluctuations in the size of EC-CFUs and the proportion of single cells. Furthermore, the ability of EPCs to migrate among themselves to form EC-CFUs varies, and the rate of EPCs differentiation and proliferation can significantly affect the number of EC-CFUs [70]. Nonetheless, flow cytometry analysis of CEPCs remains the gold standard for EPCs evaluation, despite the fact that the antigenic phenotype of these cells is still debatable and may not directly match their clonogenic potential. Next, our review will focus on the use of flow cytometry to assess CEPCs of various phenotypes.

## 4. EPCs in Chronic PA

**Figure 1:**
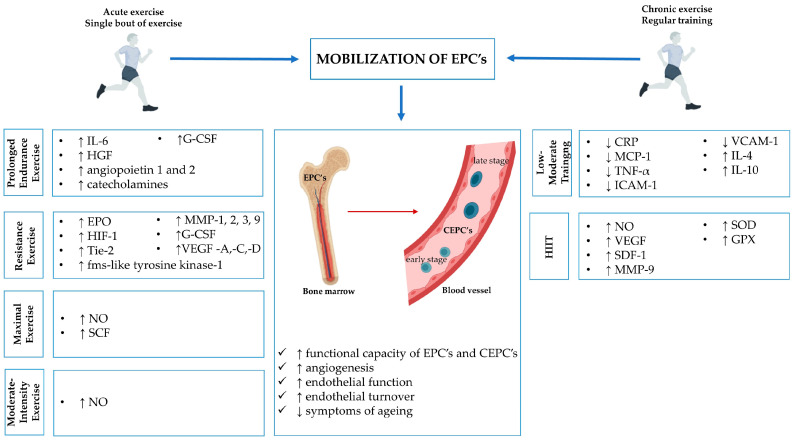
EPCs mobilization after acute and chronic exercise. IL-4,-6,-10, interleukin-4 (-6,-10); HGF, hepatocyte growth factor; G-CSF, granulocyte colony-stimulating factor; EPO, erythropoietin; HIF-1, hypoxia-induced factor-1; MMP-9, matrix metalloproteins 9; Tie-2, tunica interna endothelial cell kinase; VEGF-A (-C, -D), vascular endothelial growth factor -A (-C, -D); NO, nitric oxide; SCF, stem cell factor; MCP-1, monocyte chemoattractant protein-1; SDF-1, stromal cell-derived factor-1; CRP, C-reactive protein; TNF-α, tumor necrosis factor-α; ICAM-1, intercellular adhesion molecule 1; VCAM-1, vascular cell adhesion molecule 1; SOD, superoxide dismutase; GPX, glutathione peroxidase; EPCs, endothelial progenitor cells; CEPCs, circulating endothelial progenitor cells; ↑, increase; ↓, decrease.

Physical activity is performed for various reasons, including developing athletic skills, losing weight, increasing muscle strength, and fostering enjoyment [71]. The World Health Organisation recommends that adults aged 18–64 years perform at least 150–300 min of moderate-intensity aerobic PA, at least 75–150 min of vigorous-intensity aerobic PA, or an equivalent combination of moderate- and vigorous-intensity aerobic PA throughout the week for substantial health benefits [72]. PA is also an important method of improving vascular function in healthy individuals, thus promoting cardiovascular health and preventing vascular diseases in which EPCs play a significant role [71]. The regular practice of PA contributes to an approximately 30% reduction in mortality due to cardiovascular disease [26]. Figure 1 summarises the benefits of different types of PA (acute and chronic exercise) on the mobilization and functional capacity of EPCs.

The first study demonstrating the positive effect of PA on the mobilisation of EPCs involved a mouse model and was performed by Laufs et al. in 2004 [73]. A few years later, their findings were confirmed by another research group who performed a human study showing that the number and activity of CEPCs in 10 older and 10 younger healthy sedentary men increased after 3 months of regular PA. Interestingly, the number and activity of CEPCs showed a greater increase in the older group of men [74]. Table 2 summarises the data described in this section.

Since the performance of these studies, accumulating evidence has indicated that different types of activity (chronic and acute) have different effects on EPCs [83]. It is important to highlight how EPCs mobilisation is affected by various activity parameters, such as the mode (e.g., endurance or resistance training), duration (how long the activity is maintained during each individual session), and frequency and intensity (e.g., low intensity sustained for a long duration, moderate/high intensity sustained for a shorter duration, or very high intensity sustained for intermittent durations) [83].

Chronic activity is characterised by a very demanding PA routine that may involve intense activity on a daily basis [71]. The PA can be repeated over the short term (4–12 weeks) or long term (>12 weeks) [24]. Both types of chronic training are known to improve endothelial function in both healthy individuals and patients with cardiovascular disease [84]. Notably, the physiological responses to acute and long-term mobilisation of EPCs during exercise depend mostly on the type and dose of training: low intensity [<40% maximal oxygen consumption (VO2max)], moderate intensity (40–69% VO2max), vigorous intensity (70–90% VO2max), or very high intensity (>90% VO2max). In general, studies on the effect of chronic activity on CEPCs counts have mainly focused on patients with cardiovascular disease, including coronary artery disease, heart failure, and peripheral artery disease [84].

Few studies have addressed the relationship between chronic activity training and changes in the number and function of EPCs in healthy children [75,76,77,85]. Compared with adults, children tend to be more open-minded regarding changes in lifestyle. This suggests that early adaptation to regular PA may result in the long-lasting modification of personality traits toward an active lifestyle, at least in some children [75]. Walther et al. examined the effects of regular PA on the number and function of EPCs in sixth-grade high school students. Their study involved a total of 108 children with a mean age of 12.0 ± 0.1 years. The intervention group comprised 50 children (24 boys, 26 girls) who were assigned to one 45-min period of PA per school day. The control group comprised 42 children (18 boys, 24 girls) who were given 2 h of physical education per week. The remaining 16 children (12 boys, four girls) attended a class focusing on competitive sports and physical education, received 12 h of high-level exercise sessions per week, and frequently participated in competitive sporting events. The number and function of EPCs was evaluated after one school year. The study showed that the PA intervention was successful in increasing the EPCs count but failed to increase the migratory capacity of these cells. Notably, the highest EPCs count (CD34+/KDR+) was observed in the children attending the intervention class, and the difference compared with the control group was statistically significant [75].

In another study by Souza et al., EPCs and EPC-CFUs were evaluated in a group of children before and after moderate to vigorous PA [76]. EPCs were quantified as CD34+/CD133+/KDR+ cells. Forty children (22 boys, 18 girls) aged 7–11 years participated in a 10-week moderate to vigorous PA programme (duration, 45 min; intensity, 75–85% of heart rate reserve; frequency, four sessions/week) [76]. After the 10-week programme, the circulating/functional capacity of CD34+/CD133+/KDR+ cells had significantly increased. One year later, this research group used the same protocol in a study of low-birth-weight children. In this second study, 35 children aged 6–11 years participated in a 10-week PA programme. The combination of low birth weight and the PA programme had a significant interaction effect on the numbers of CEPCs and EPC-CFUs [77]. Ten weeks of physical intervention resulted in an almost two-fold increase in the number of EPCs in low-birth-weight children, and a similar trend was observed in the number of EPC-CFUs.

Studies have also been performed to investigate the effects of chronic activity on CEPCs in healthy adults [78,79,80]. Bittencourt et al. compared the EPCs counts between professional runners and healthy controls. In total, 25 half-marathon runners and 24 age- and sex-matched healthy controls were included in the study [78]. The exercise training programme in the group of athletes involved two long-distance running sessions every day (15 km in the morning and 10 km in the afternoon) and intensive training twice a week (100 to 1000 m bursts of intense running, repeated numerous times). EPCs (CD34+/KDR+, CD133+/KDR+, and CD34+/CD133+ cells) were quantified by flow cytometry. After the training programme, the CD34+/KDR+ and CD133+/KDR+ EPCs counts were higher in the athletes than in the controls, whereas the CD34+/CD133+ EPC count was not different between the two groups [78]. Another trial assessed the effect of a personalised PA programme on circulating progenitor cells (CPCs) (defined as CD34+, CD133+, and CD34+/CD133+ cells) and EPCs (defined as CD34+/KDR+, CD133+/KDR+, and CD34+/CD133+/KDR+ cells) in 80 overweight and obese subjects [79]. The participants comprised 40 men and 40 women aged 24–69 years with a body mass index of >25 kg/m^2^ and no other established cardiovascular risk factors (i.e., hypertension, diabetes, dyslipidaemia, or a family history of cardiovascular disease). At the beginning of the trial, the participants were instructed to attend a PA programme for 3 months. The training protocol consisted of a 45-min session of aerobic exercise (brisk walking or moderate running) three times per week [79]. At the end of the intervention programme, the population was divided into two groups: compliant individuals (*n* = 47) and non-compliant individuals (*n* = 33). Compliant individuals were defined as those who self-reported adherence to the whole training programme. After 3 months of PA, the circulating levels of CPCs and EPCs showed an increasing trend in the compliant group, reaching statistical significance for all three types of EPCs. By contrast, the non-compliant group showed no significant increase in the tested cell populations [79]. Interestingly, Rakobowchuk et al. indicated that interval training did not alter the CEPCs counts as defined by CD34+, CD133+, and CD309+/KDR+ antigen expression [80]. Their study involved 20 healthy volunteers who underwent training for 6 weeks. The training involved either moderate (*n* = 9) or heavy (*n* = 11) metabolic stress interval activity, with the two groups matched for total work and activity duration. It was hypothesised that the lack of change in the CEPCs counts was related to the small number of participants.

It is well documented that older age is typically associated with lower CEPC counts [29,74,82]. In terms of training characteristics, even a short-duration (10-day) but moderate-intensity (daily aerobic activity at 70% VO2max) continuous training activity programme can increase CEPCs counts in sedentary older individuals [81]. Xia et al. showed that the circulating CD34+/KDR+ and KDR+/CD133+ cell counts increased in a group of 22 elderly men after 30 min of daily training performed 3 days per week for 12 weeks [29]. By contrast, Thijssen et al. showed that in both young and older sedentary men, 8 weeks of endurance training did not affect the baseline or activity-induced EPCs counts. Notably, EPCs were defined as CD34+/VEGFR-2+ cells in their study [82].

Sex-specific differences are known to be present in the quantitative and functional characteristics of CEPCs [86]. However, there is no evidence for sex-specific chronic activity-induced changes in CEPCs. This knowledge gap might serve as a guide for future research.

## 5. EPCs in Acute PA

Studies examining the effects of acute activity on CEPCs include various types of training: endurance training, maximal training, moderate-intensity continuous training (MICON), continuous exercise (CONT), muscular endurance resistance exercise (MERE), and high-intensity interval training (HIIT). Different patterns of changes in EPCs mobilization occur under the influence of various types of acute training. These may be accompanied by changes in various mediators of angiogenesis, markers of muscle damage, and inflammatory factors.

Möbius-Winkler et al. noted a correlation between EPCs mobilization and increased VEGF levels after acute activity. Four hours of cycling at 70% of the individual anaerobic threshold caused an increase in the EPCs count (at a maximum of 240 min after the start of PA) accompanied by an increase in the interleukin-6 concentration (at a maximum of 30 min after the test) and VEGF concentration (at a maximum of 10 min after the start of activity). All observed changes returned to baseline values at least 24 h after the completion of activity. The authors considered the mobilisation of EPCs to be associated with the increased VEGF concentration, which can be viewed as a physiological mechanism aimed at maintaining an intact EC layer [87].

The MERE exercise protocol has also been shown to impact the mobilisation of EPCs and the release of angiogenic factors such as VEGF-A, VEGF-C, VEGF-D, MMP-1, MMP-2, MMP-3, MMP-9, Tie-2, and soluble fms-like tyrosine kinase-1. The MERE protocol, which includes three circuits of six exercises with 15 repetitions of each exercise, is performed on a resistance machine. One study showed that after 24 h, all changed parameters returned to pre-exercise levels [88].

Strömberg et al. recruited 20 men with a moderately active lifestyle and divided them into two groups. Each participant performed 60 min of PA on an electrodynamically loaded cycle ergometer. The first 20 min of activity was performed at a speed of 60 rpm and a work intensity corresponding to 50% of VO2max. For the next 40 min, the work rate was increased to a workload corresponding to 65% of VO2max. Each participant’s perceived effort was measured using the Borg Rating of Perceived Exertion scale, and the load was then modified accordingly. In the first group, venous blood was collected before, immediately after, 30 min after, and 2 h after activity. In the second group, a biopsy of the vastus lateralis muscle was performed before and 2 h after activity, and venous blood was collected at rest and immediately after, 30 min after, and 2 h after activity. Cytometric analysis of circulating cells with known remodelling properties showed a significant increase in the levels of monocyte subpopulations and EPCs after a single episode of acute PA. The increase in the numbers of monocytes and EPCs was accompanied by the increased release of VEGF-A and endothelial adhesion molecules such as intercellular adhesion molecule-1, vascular cell adhesion molecule-1, and E-selectin in muscle tissue, which may be important in the remodelling of skeletal muscles [89].

One study analysed the mobilisation of HPCs and EPCs in response to endurance, resistance, and eccentric activity and examined the relationship of these cells with markers of muscle damage and inflammation. The study recruited 36 healthy men aged 21–29 years, and the men were divided into three groups: one group performed a concentric endurance test, one group performed a resistance activity test, and one group performed an eccentric endurance test. None of the participants had performed any exercise training for at least 2 days prior to the activity protocols. Blood samples were collected immediately before, immediately after, 1 h after, 3 h after, and 24 h after all exercise tests (an additional time point of 48 h was added for the resistance and eccentric endurance test groups because a continuous increase in some of the tested parameters was observed). After the endurance test, HPCs and EPCs exhibited an immediate and short-term increase, whereas the eccentric endurance test induced a long-term increase in HPCs (up to 24 h) and EPCs (up to 48 h). In the resistance exercise group, an increase in HPCs was found only 3 h after PA. Moreover, a correlation was observed between EPCs mobilization and the systemic G-CSF concentration, as well as HPC count and creatine kinase concentration. These findings suggest that a specific pattern of progenitor cell mobilisation is induced depending on the type of acute physical activity performed, and the regulation of this mobilisation may be mediated by factors such as G-CSF and creatine kinase [90].

The increase in the EPCs count after acute PA may be related not only to angiogenic factors but also to the lipid profile. Van Craenenbroeck et al. observed increased EPCs mobilization under short-term acute activity, and this was accompanied by an increase in VEGF but not nitric oxide secretion. Participants with higher levels of low-density lipoprotein cholesterol, high-density lipoprotein cholesterol, and total cholesterol showed a more intense increase in the EPCs count. These results suggest that higher lipid levels, which ensure a pro-oxidant vascular environment, provide a stronger stimulus for EPCs mobilization in healthy individuals [91].

Yang et al. confirmed the theory that EPCs mobilization induced by acute PA is also related to nitric oxide. Sixteen healthy men participated in a modified protocol of intense exercise on a Bruce treadmill, and the test was ended when the men declared significant physical exhaustion. Venous blood samples were collected before PA and 30 min after the end of the test. In the linear regression analysis, the researchers observed a significant relationship between elevated plasma nitric oxide levels and increased numbers of CEPCs. This finding suggests that nitric oxide plays an important role in the activity-induced regulation of CEPCs [92].

Bonsignore et al. examined the post-activity mobilisation of endothelial progenitors in healthy, trained individuals according to the type of activity performed. Two types of PA were tested: endurance (marathon running) and maximal (1500 m run at maximum speed at 75% of the maximum predicted heart rate [approximately 130 beats/min]). The results showed that maximal activity was associated with the release of both HPCs and EPCs. However, a different reaction was observed after endurance training: mobilisation of CEPCs and a reduced number of HPCs. Activity-induced progenitor cell release has been linked to the release of growth factors, which may help to understand the mechanisms involved in progenitor mobilisation. A dose–response effect was observed, with higher plasma concentrations of interleukin-6, hepatocyte growth factor, and angiopoietin 1 and 2 after the marathon than after the 1500 m run. However, the concentration of stem cell factor increased similarly in both testing protocols [93].

Krüger et al. also compared two types of acute activity: high-intensity interval training and continuous activity. After both activity protocols, leucocytosis occurred and persisted for up to 3 h. Both types of activity effectively mobilised HPCs and EPCs, with the increase in HPCs occurring immediately after high-intensity interval training and 3 h after continuous activity. However, EPCs mobilization occurred immediately after activity [94].

Acute activity transiently increases the number of circulating naïve T-cell, effector memory T-cell, and EPCs subpopulations in both men and women. Greater increases in these cell counts have been seen after high-intensity sprint exercise than after long-term, continuous endurance exercise [95].

Laufs et al. examined the duration and intensity of activity at which EPCs were mobilised into the circulation. They concluded that 30 min of moderate (approximately 68% of VO2max) and intense (approximately 82% of VO2max) activity increased the plasma EPCs count. The maximum increase in CEPCs was observed after 10–30 min of intense running. Ten minutes of moderate-intensity running was unable to produce such an effect [96].

Adams et al. reported interesting findings regarding the effect of marathon running on the release of HPCs and EPCs. They recruited healthy men who were older than 50 years and had completed at least five full-distance marathon races in the previous 3 years. Participation in a marathon was found to trigger an inflammatory reaction as manifested by a significant increase in the number of leucocytes immediately after the run, whereas the number of CPCs (CD34+, CD133+, and CD117+ cells) significantly decreased. Interestingly, no significant changes in the number of EPCs were detected. Thus, EPCs mobilization might occur later after marathon running. Notably, in the above study, blood samples were collected only immediately after PA [97].

Chang et al. assessed the effect of acute activity on the mobilisation of EPCs from the bone marrow into the peripheral blood in healthy volunteers. PA in the form of a 30-min run at a heart rate of >140 beats/minute was found to be sufficient to mobilise CEPCs, and this mobilisation was accompanied by an increase in the plasma concentration of SDF-1α. These increases were observed both immediately and 24 h after the test [37].

CACs include several subpopulations of PBMCs that express endothelium-specific markers. One study was performed to examine whether acute physical activity affects the CACs count. The study focused on a population of young, healthy women and men aged 18–40 years who participated in a treadmill exercise protocol. The treadmill was operated at a constant speed, and the incline was increased by 2.5% every 2 min until exhaustion. Blood samples were collected from the volunteers before activity and immediately after the test. Maximum activity increased the CD31+ cells, CD31+ cells, CD62E+ cells, CD14+/CD31+ cells, and CD34+/VEGFR-2+ PBMCs by 40%, 29%, 33%, 14%, and 33%, respectively, whereas CD3+/CD31+ and CD3+ cells remained unchanged by exercise. These results correspond to other scientific reports in the context of mobilisation of EPCs (designated as CD34+/VEGFR-2+ cells) induced by acute, maximal PA [98].

Niemiro et al. obtained different results in regard to post-exercise EPCs mobilization. After the participants performed a 60-min maximum exercise test protocol on a motorised treadmill according to the modified Balke protocol, they showed an increase in the total CPCs population, while the EPCs and MSCs populations did not change [60]. These findings may indicate that mobilisation of CPCs (haematopoietic stem/progenitor cells, haematopoietic stem cells, EPCs, and MSCs) is atypical in young adults.

The effect of acute occlusion activity on EPCs mobilization has also been assessed. Blood flow-restricting activity was hypothesised to promote activity-induced EPCs mobilization due to the severity of local tissue hypoxia. The analysis involved men aged 18–30 years who performed two trials of unilateral knee extensor exercises with occlusion. No significant changes in the numbers of EPCs and HPCs were observed during the post- activity recovery period. In the control group, however, in which the same exercise protocol was performed without occlusion, an increase in the EPCs count was observed 30 min after the test. The lack of significant changes in the occlusion exercise group may have been due to the limited blood collection schedule and insufficient training stimulus; significant changes in the number of EPCs might occur later after training [99].

Age may be another factor that determines the mobilisation of progenitor cells. The progenitor cell count has been found to decrease with age. In a study by Ross et al., post- activity EPCs mobilization was more intense in younger people than in older people. Additionally, compared with younger adults, older adults showed reduced naïve T-cell flow into the blood in response to an acute activity stressor. This is consistent with other scientific findings [100].

Few scientific reports have focused on the changes in EPCs that occur under the influence of PA only in women. Ribeiro et al. examined 38 women who performed resistance training (three sets of four exercises at 12 repetitions each) at an intensity of 60%, 70%, or 80% of one repetition maximum. Blood was collected from the volunteers at the beginning of the study and immediately after, 6 h after, and 24 h after activity. The researchers noted a significant increase in the mobilisation of EPCs and angiogenic factors such as VEGF, hypoxia-induced factor-1α, and erythropoietin after activity. The change in EPCs from baseline was greatest in the 80% intensity session group and peaked 6 h after activity. Thus, the mobilisation of progenitor cells and angiogenic factors in young women seems to depend on the activity intensity, which is consistent with the findings of studies involving men [101].

In another study, researchers examined whether interval or continuous exercise had an impact on endothelial function and the number and function of CACs in postmenopausal women. The researchers found no significant differences in the number of CACs, including EPCs, after 30 min of acute moderate-intensity continuous training, interval exercise training, or heavy interval training sessions on a cycle ergometer. However, other research has shown that acute interval exercise increases the ability of CACs to form colonies, which may increase the capacity for vascular repair in postmenopausal women [102].

## 6. Future Perspectives

ECs line the luminal surface of blood vessels and play an important role in vascular homeostasis. Additionally, these cells have the ability to modulate the repair and growth of blood vessels. Therefore, they constitute an important component in the prevention and development of CVDs. CEPCs contribute to vascular repair and regeneration. Current research indicates that CEPC quantity in peripheral blood may be clinically useful as an early marker of CVD risk. The blunted post-activity CEPCs response (vascular repair) in patients was noted. In advanced CVD patients, the number of CEPCs is reduced, potentially due to the myelosuppressive effects of TNF-α, and the treatment with TNF-α inhibitor has shown to significantly increase CEPC levels in rheumatoid arthritis patients [103]. Inflammatory processes suppress stem cell turnover and function. However, inflammation indirectly increases the demand for endothelial repair and turnover. Thus, several strategies that are well-known inflammatory modulators may be applied, such as diet or exercise [104]. The level of CEPCs can be a good indicator in monitoring the fatigue in athletes (overtraining), the effectiveness of training, and the capacity of vascular regeneration. Undoubtedly, further research is required to investigate the interaction between the CEPCs and inflammatory signals with specific emphasis on the mechanisms of inflammation.

Vascular ageing is associated with changes in the mechanical and structural properties of the vascular wall. Together with the reorganisation of the extracellular matrix it leads to endothelial dysfunction. Physical intervention improves the re-endothelialisation and increases CXCR4 protein expression and JAK-2 phosphorylation in EPCs. New strategies of PA should be explored as lifestyle intervention strategies to promote vascular health and endothelial turnover in the aging population.

Moreover, what is greatly interesting is that EPCs interact and communicate with other cell types. This specific relation may be attributed to direct cell-to-cell contact, secretion of cytokines, chemokines, growth factors, or exosomal vesicles. It is known that EPCs’ relation with macrophages in the brain plays a critical role in regulating blood–brain barrier function, vascular perfusion, and inflammation [105]. Similar close relations exist in the maintenance of muscular biology/regeneration and bone homeostasis [106,107,108]. Recent evidence shows that extracellular vesicles released from EPCs carry, among various other substances, particles of miRNA (EPC-EV-derived miRNAs) that have significant cardioprotective properties [109]. Furthermore, exercise modifies the uptake efficiency of exosomes by neighbouring neural cells and reduces ROS production in hypertensive transgenic mice [110].

Training-induced impairment of endothelial function may have detrimental effects on vascular health. In particular circumstances, PA in athletes may lead to adverse changes in endothelial status that may have significant long-term effects on performance and progress. Therefore, endothelial condition should be regularly monitored in the time-course of the training process to minimalise vascular health risk in athletes.

With advancing age, the secretion of vasoconstrictor factors increases, which leads to vasodilatory dysfunction and significant endothelial damage. Moreover, the loss of elastic fibres and increased collagen production reduces vascular elasticity. This condition results in inflammation and endothelial damage. Many traditional risk factors that have been linked to CVDs are also accentuated in the advanced age population. Chronic aerobic activity is intended to prevent and/or reverse age-related endothelial dysfunction by lowering systemic inflammation and increasing the number and regenerative capacity of CEPCs. Regular aerobic activity is considered an optimal strategy and may be an excellent tool to prevent or reverse age-related degenerative vascular changes.

EPCs are precursor cells of ECs, which can differentiate into vascular ECs, and protect from tissue ischemia and endothelial dysfunction. Therefore, they may be a potential source of cells in transplantation strategies. In clinical trials, it has been proven that EPCs play both an important role in vasculogenesis and have a positive paracrine effect. The first results of clinical EPCs application in cardiovascular, neurological, and respiratory diseases are promising. Although no significant side effects of EPCs use were observed, further studies are obligatory to confirm the long-term safety and effectiveness of these methods and to assess possible risks.

## 7. Summary

Physical activity is undoubtedly an excellent non-pharmacological tool with which to reduce inflammation and improve endothelial function, thus counteracting unfavourable processes occurring in the cardiovascular system. The effects of physical activity can be observed and measured after both short- and long-term PA. Almost 30 years have passed since the first identification and isolation of EPCs by Asahara et al. Nevertheless, the origin, heterogeneity, and biology of EPCs make it difficult to recommend clear criteria that can be used for their evaluation [12]. The selection of an EPCs assessment method and evaluation of the phenotype of these cells should be tailored to the mechanisms being studied, because the possibility of identifying these cells is strictly correlated with the techniques used to evaluate their surface markers. EPCs are a diverse subpopulation of CD34+ cells, and further research is needed to refine the definition of these rare cells in both the circulation and peripheral tissues. Also, in the context of regenerative medicine and tissue engineering, EPCs are an important and promising field of research.

## Figures and Tables

**Table 1:** Phenotypes used to identify EPCs and CECs by flow cytometry.

Cell Phenotype	Origin of Cells	Reference
CD34^+^	EPCs	[56,57]
CD34^+^/CD45^dim/neg^/KDR^+^	EPCs	
CD34^+^/CD45^neg^/CD146^+^ CD45^neg^/CD34^+^/CD31^+^/CD36^+^	CECs CECs (microvascular origin)	[57]
CD34^+^/CD45^−^	EPCs	[58]
CD34^+^/CD133^+^	CPCs	[59]
CD45^−^/CD34^+^/CD31^+^	EPCs	[60]
CD34^+^/CD45^−^/CD133^+^ CD34^+^/CD45^−^/CD133^+^/VEGFR-2^+^ CD34^+^/CD133^+^/VEGFR-2^+^	EPCs	[61]
CD34^+^/CD45^−^/CD133^−^ CD34^+^/CD45^−^/CD133^−^/VEGFR-2^+^	CECs	[61]
CD45^dim^/CD34^+^/CD133^+^/VEGFR-2^+^	EPCs	[62]
CD34^+^/VEGFR-2^+^ CD34^+^/VEGFR-2/CD3^−^	EPCs	[55]
CD45^−^/CD146^+^/CD34^+^/VEGFR-2^+^	Late EPCs	[63]
CD34^+^/CD144^+^/CD3^−^	CEPCs	[64]
CD45^dim/neg^/CD34^+^/CD146^+^/CD133^+^ CD45^dim/neg^/CD34^+^/CD146^+^/CD133^−^	EPCs CECs	[65]

EPCs, endothelial progenitor cells; CECs, circulating endothelial cells; CPCs, circulating progenitor cells; CEPCs, circulating endothelial progenitor cells; VEGFR, vascular endothelial growth factor receptor; KDR, kinase domain region.

**Table 2:** Summary of studies examining the effects of chronic exercise on EPCs levels.

Number of Participants/Gender	Age	Parameters of PA	Results	References
108 children	12.0 ± 0.1 years old	intervention group physical exercise (45 min) per school day;	↑ amount and function of EPCs in the intervention class;	[75]
(54 boys and 54 girls)		control group 2 h of physical education per week	↑ amount and function of EPCs in the intervention class	
40 children (22 boys and 18 girls)	7–11 years old	10-week (MVPA) programme (duration: 45 min; intensity: 75–85% of heart rate reserve; frequency: 4 sessions/wk)	↑ circulating functional capacity of CD34+/CD133+/KDR+ cells	[76]
35 children	6–11 years old	10-week (MVPA) programme (duration: 45 min; intensity: 75–85% of heart rate reserve; frequency: 4 sessions/wk)	2-fold increase in the number of EPCs	[77]
49 women and men	-	intervention group 2 long-distance running sessions every day: 15 km in the morning and 10 km in the afternoon, and intensive training (100–1000 m shots, repeated many times) twice a week	↑ CD34+/KDR+ EPCs, CD133+/KDR+ EPCs, and CD34+/CD133+ EPCs were not different in athletes	[78]
80 subjects (40 men, 40 women)	24–69 years old	45-min session of aerobic exercise (walking briskly or moderate running) 3 times per week for 3 months	↑ CPC: CD34+, CD133+, and CD34+/CD133+; ↑ EPC: CD34+KDR+, CD133+KDR+, and CD34+CD133+KDR+ in compliant group	[79]
20 volunteers (7 men, 13 women)	23.7 ± 3.4 years old	6 weeks, training involved either moderate (MSIT; *n* = 9) or heavy metabolic stress (HSIT; *n* = 11) interval exercise	CD34+, CD133+, and CD309/KDR+ did not alter	[80]
11 adults (4 men, 7 women)	61 ± 2 years of age	60 min of aerobic exercise at 70% maximal oxygen consumption for 10 consecutive days	CD34+/KDR+ number increased 104% and KDR+ number increased 151%	[81]
20 men	young group 21–33 years old and old group 59–72 years old	regular aerobic exercise ≥4 times/wk, ≥30 min/ session for 3 months	↑ CD34+, KDR+ in young and old men	[74]
22 men	67.8 ± 3.38 years old	30 min daily for 3 days per week for a period of 12 weeks	↑ CD34+/KDR+ and KDR+/CD133+ number of cells	[29]
16 men	young group 18–28 years old and old group 67–76 years old	8 weeks, endurance training	CD34+/VEGFR-2+ did not alter	[82]

EPCs, endothelial progenitor cells; MVPA program, moderate to vigorous physical activity program; ↑, significant increase; CPCs, circulating progenitor cells.
